# Intracystic Papillary Carcinoma of the Breast: Report of Three Cases and Literature Review

**DOI:** 10.1155/2012/979563

**Published:** 2012-02-14

**Authors:** Yassir Ait Benkaddour, Sawsane El Hasnaoui, Karima Fichtali, Bouchra Fakhir, Hicham Jalal, Mouna Kouchani, Abderrahim Aboulfalah, Hassan Abbassi

**Affiliations:** ^1^Department of Obstetrics and Gynecology, University Hospital of Marrakesh, Cadi Ayyad University, 13 bis, Lot Riyad Salam, 40100 Marrakesh, Morocco; ^2^Department of Radiology, University Hospital of Marrakesh, Cadi Ayyad University, 40000 Marrakesh, Morocco; ^3^Department of Oncology, University Hospital of Marrakesh, Cadi Ayyad University, 40000 Marrakesh, Morocco

## Abstract

Intracystic papillary carcinoma is a rare malignant tumor of the breast. It occurs communally in postmenopausal women. Clinically it can be asymptomatic or manifested by a breast mass or a nipple discharge. On imaging intracystic papillary carcinoma has usually benign features. Pathologic diagnosis can be difficult at classical histological examination and identification of myoepithelial cells layer by immunohistochemical study can be useful. In the majority of cases of pure intracystic papillary carcinoma, conservative management is possible. Adjuvant therapy is still controversial and prognosis is excellent. We report three cases of intracystic papillary carcinoma diagnosed on immunohistochemical examination and managed with conservative surgery.

## 1. Introduction

Intracystic papillary carcinoma is an uncommon breast disease, constituting 0.5% to 1% of all breast cancers [1]. Papillary carcinomas are classified histologically into intraductal and intracystic papillary carcinoma [2]. Intracystic papillary carcinomas (IPCs) are further divided into pure form or associated to a ductal carcinoma in situ (DCIS) or invasive carcinoma [1].

Clinical and radiologic manifestations of IPC are not specific. On ultrasonography, it can be a pure cyst, a mixed image, or a solid mass.

The recent use of immunohistochemical identification of myoepithelial cells layer (MEC) of the cysts seems to enhance pathologic diagnosis performance but its influence on prognosis is to be determined.

Therapeutic management of IPC is also still controversial. Endocrine therapy and radiation are used by many centers but evidence of their role in prognosis improvement is still lacking.

## 2. Case Report 1

A 58-year-old woman presented with a mass in the upper internal quadrant of the left breast, without pain or nipple discharge.

Physical examination revealed a 2 cm mass, non-well-defined, fixed and firm, nonpainful, and without skin changes.

**Figure 1:**
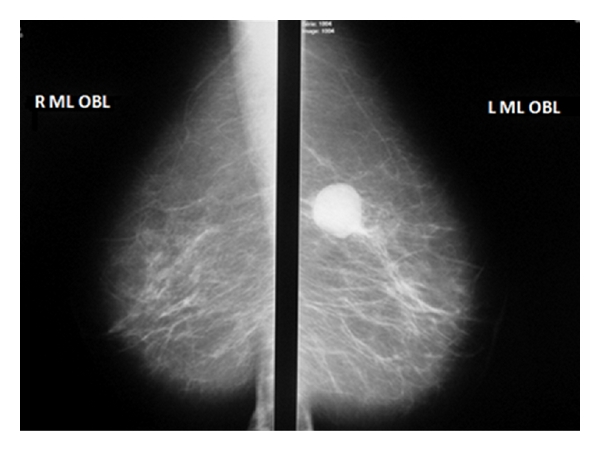
Mediolateral oblique mammogram which shows a soft-tissue mass, well limited in the left breast.

**Figure 2:**
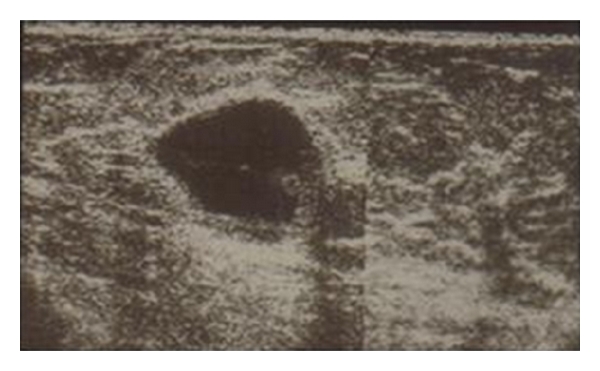
Ultrasonography showed a cystic mass of 27 mm, with posterior acoustic enhancement, and a solid component.

Mammography showed an oval opacity of the upper internal quadrant with no calcifications (Figure 1). Ultrasonography showed a cystic mass measuring 27 mm, with posterior acoustic enhancement and a little solid component (Figure 2). Surgical excision was performed.

At the pathologic examination, the tumor was a cyst containing a solid grey material.

Histological examination revealed an intracystic papilloma with atypical hyperplasia. Immunohistochemical study did not found neither anti-p63 nor anti-cytokeratin 5/6 antibodies and conclude to an intracystic papillary carcinoma. Surgical margins were negative.

The patient received an adjuvant treatment based on endocrine therapy and breast radiation. There was no recurrence at 13 months of follow-up.

## 3. Case Report 2

 A 40-year-old patient, with a history of surgery for an intraductal papilloma of the left breast three years ago, presented with a mass of the same breast.

Physical examination revealed a 3 cm, well-circumscribed mass, firm and painless, in the lower internal quadrant of the left breast. There were no axillary nodes.

**Figure 3:**
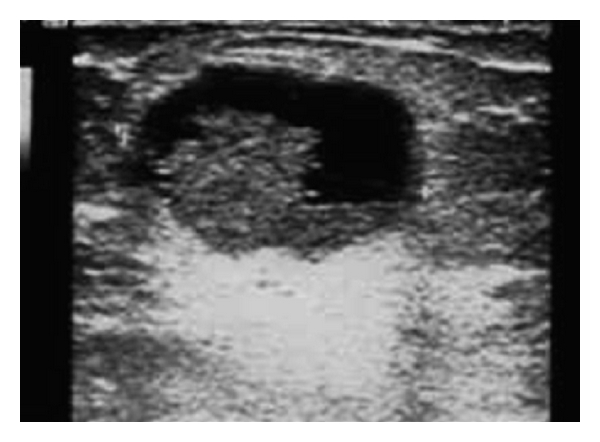
Heterogeneous mass with tissular and cystic components.

On sonography the mass was heterogeneous with solid and cystic components, measuring 25 mm (Figure 3). These finding suggested a benign tumor and surgical excision was performed.

Mammography shows a homogeneous lobulated opacity of the lower and internal quadrant of the left breast without calcifications.

Histological examination revealed an intracystic papillary carcinoma with positive surgical margins. Immunohistochemical study shows absence of MEC layer and confirmed the diagnosis.

A conservative surgical reexcision was performed, and surgical margins were free of disease. No adjuvant radiation or endocrine therapy was administered.

At the follow-up of 9 months, there was no recurrence.

## 4. Case Report 3

A 70-year-old patient presented with a breast lump. Examination found a painless, well-circumscribed mass measuring 4 cm, without axillary nodes. 

Mammography has shown a round regular opacity, without calcifications. Sonography revealed a pure cystic mass.

**Figure 4:**
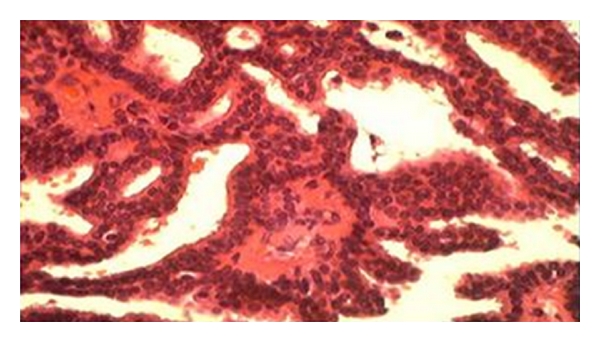
Papillae with mild cellular atypia.

Surgical excision was performed with frozen section examination. It revealed an intracystic papilloma with atypia (Figure 4). The definitive pathological examination confirmed this finding. Immunohistochemical study showed a complete negativity of P63 compatible with the diagnosis of intracystic papillary carcinoma. Surgical margins were free of disease and the patient has not received adjuvant treatment. The patient presented a recurrence one year after the first surgery and was managed by a second conservative surgery.

## 5. Discussion

Papillary carcinoma of the breast is a rare malignant tumor, constituting 1-2% of all breast carcinomas in women [3]. It is distinguished by the papillary structural design: proliferation characterized by finger-like projections or fronds composed of central fibrovascular cores covered by epithelium, without myoepithelial cell layer (which differentiate between benign and malignant papillary lesion). It can be divided into invasive and noninvasive forms. Noninvasive papillary carcinomas are further subdivided into two subtypes: a diffuse form, the papillary variant of ductal carcinoma in situ, and a localized form, intracystic papillary carcinoma [1, 4]. This localized form, IPC, describes a solitary tumor in an encysted or dilated duct.

The intracystic papillary carcinoma is more frequently found among postmenopausal women with an average age between 55 and 67 years old [5], many cases were also described in the male population in the literature, and it is the second men's breast cancer [6–8].

Clinically, it frequently presents as a benign-like mass. The tumor can also manifest with a bloody nipple discharge, and in some cases, it can be asymptomatic and revealed by systematic mammography. Axillaries nodes are infrequent [4, 6].

At the mammography, the intracystic papillary carcinoma appears as a round, ovular, or lobulated opacity. The margins of the mass are usually circumscribed but may be obscured or indistinct by places testifying inflammation or invasion [4, 6]. There are no speculations. The differential diagnosis on mammographic appearance includes a hematoma, invasive ductal carcinoma, and colloid or medullar carcinoma, benign cyst, or adenofibroma [9].

On ultrasonography, the lesion might have an indistinct border or microlobulation, which might suggest that it is malignancy, it is often complex in echo texture and shows both cystic (anechogenic) and solid (echogenic) components, the cystic portion may contain septation, with solid papillary mass projecting into the cystic lumen, internal echoes may be identified, and they often related to spontaneous hemorrhage within the tumor in the cyst [2, 10, 11]. Color Doppler is helpful to demonstrate the intramural blood flow within the solid component of the mass.

An important size of the intracystic vegetation, the heterogeneous echo texture, and irregular border of the solid papillary mass are criteria for malignancy suspicion.

The magnetic resonance imaging (MRI) is sensitive but not specific in detecting papillary tumors. It is useful in case of multiple papillomatosis, but without any interest to distinguish benign and malignant lesions [2, 8].

The combination of a residual palpable mass and a frankly bloody aspirate at the fine needle aspiration is a strongest indicator of carcinoma.

Core needle biopsy of the intracystic mass with ultrasonographic guidance has been used by many authors for distinguishing benign from malignant papillary lesions, but it has a low accuracy for distinguishing between in situ or invasive papillary carcinoma because the site of biopsy is generally central while the invasion is usually found in the periphery of the tumor [4, 6].

Surgical excision is recommended after core-needle biopsy if there is atypia, high-risk lesion, positivity for malignancy, or imaging-histological discordance. Biopsy excision is often performed directly when papillary carcinoma is suggested by sonography or mammography.

We performed surgical excision for all our patients because of patient's age and the presence of a solid component. We think that, in these cases, the sensitivity of core needle biopsy is low and it does not allow skipping the surgical excision anyway.

For Tomonori et al. [12], also, excisional biopsy is necessary because IPC is more difficult to diagnose than common breast cancer preoperatively; fine needle aspiration and core needle biopsy are not sufficient most of the time.

Indeed, the surgical excision allows the pathologist to classify the papillary lesion by classical histological examination and especially with immunohistochemical study and to research invasion or DCIS in surroundings breast tissues, present in majority of cases [13].

Identification of a myoepithelial cell layers essentially by immunohistochemical analysis has become a key feature in distinguishing benign from malignant and in situ from invasive papillary lesions of the breast [14, 15]. In the case of IPC, there is completely a lack of myoepithelial cell layers both in the proliferating intraluminal component of the lesion and in the basal layer at the periphery, a situation similar to invasive papillary carcinoma, but with fibrous capsule surrounding it, so many authors recently describe IPC as an encapsulated low-grade invasive carcinoma and accept it as a borderline lesion in progression from in situ to invasive carcinoma [7, 13, 16].

The largest series of intracystic papillary carcinoma in the literature, with an analysis of over 900 cases of IPC, retrospectively from the data of the California Cancer Registry (CCR), reviewed from the years 1988 to 2005, demonstrate that distinguishing between in situ or invasive IPC is likely not of clinical significance because, regardless of the classification, the prognosis is excellent [7].

There are no evidence-based guidelines for treatment of IPC. There is no randomized controlled trial comparing breast conserving surgery to mastectomy. However, many case reports and retrospective studies showed excellent prognosis with conservative surgery without axillary dissection in IPC not associated to DCIS or microinvasion lesions [1, 17, 18]. 

Sentinel node biopsy may be an excellent alternative to full axillary dissection in patients with IPC and associated invasive carcinoma [1].

There is also lack of evidence about the role of adjuvant therapy. In one of the biggest series [18] we found in the literature, Fayanju et al. reviewed the management of 45 patients with IPC in order to determine factors associated with the use of adjuvant therapy. In this study, authors concluded that the most important factor determining the use of radiation and endocrine therapies is associated pathology (DCIS or microinvasion) and patients with pure IPC were less likely to undergo radiation and endocrine therapies.

Even if IPC is associated to breast DCIS or invasion, its prognosis is still excellent when treatment decisions are tailored to associated pathology [18].

In this context of lack of evidence about adjuvant therapy, we based the use of adjuvant therapy on the patient's opinion after informing them that these treatments are optional.

## 6. Conclusion

Intracystic papillary carcinoma is rare breast malignancy, with an excellent prognosis in its pure form.

Suspected at sonography in front of cyst with internal solid component, occurred to premenopausal woman, it will be confirmed by histopathology and immunohistochemical study after surgical excision.

The mainstay of treatment is surgical resection, with adjuvant therapy if associated to DCIS or invasive carcinoma.
